# Rheological properties of dry-fractionated mung bean protein and structural, textural, and rheological evaluation of meat analogues produced by high-moisture extrusion cooking

**DOI:** 10.1016/j.crfs.2023.100552

**Published:** 2023-07-21

**Authors:** Davide De Angelis, Christina Opaluwa, Antonella Pasqualone, Heike P. Karbstein, Carmine Summo

**Affiliations:** a University of Bari “Aldo Moro”, Department of Soil, Plant and Food Science (DISSPA), Via Amendola, 165/A, 70126, Bari, Italy; b Karlsruhe Institute of Technology, Institute of Process Engineering in Life Sciences, Chair of Food Process Engineering, Gotthard-Franz-Straße 3, 76131, Karlsruhe, Germany; c Fraunhofer Institute for Process Engineering and Packaging IVV, Giggenhauser Straße 35, 85354, Freising, Germany

**Keywords:** High-moisture extrusion, Plant-based protein, Closed cavity rheometer, Textural evaluation, Meat analogues, Dry-fractionated protein

## Abstract

A closed cavity rheometer was used to study the rheology of dry-fractionated mung bean protein –DFMB– (55% protein d.m.). Then, the high-moisture extrusion cooking at 40% and 50% moisture contents and different temperatures (115, 125, 135 and 145 °C) was performed, investigating the impact on structural, textural, and rheological properties of extrudates. When subjected to a temperature ramp (40–170 °C), DFMB showed an increase of G* from 70 °C, as a consequence of starch gelatinization and protein gelation. The peak, indicating the end of aggregation reactions, was at 105 °C and 110 °C for DFMB at 50% and 40% moisture content, respectively. The time sweep analysis described the protein behavior in no-shear/shear conditions, highlighting a more pronounced effect of the temperatures compared to moisture content. During the extrusion cooking, the temperature increase led to a decrease of pressure, indicating a reduction of the melt viscosity. The microstructure of the extrudates showed a more pronounced anisotropic profile when higher temperatures were applied. Hardness, chewiness, and cohesion were directly correlated with the temperature, which also affected the rheological properties of extrudates. A combination of textural and rheological analyses can offer a clear overview of the structural characteristics of meat analogues.

## Highlights

• Dry-fractionated mung bean protein can be used in meat analogues applications.
• The rheology of the protein was evaluated in extrusion relevant conditions.
• Higher extruder temperature affected pressure and microstructure of the extrudates.
• Both rheology and texture analysis allow a comprehensive extrudate evaluation.
• The texture map of extrudates revealed similarities with some commercial products.

## Graphical abstract

**Figure:**
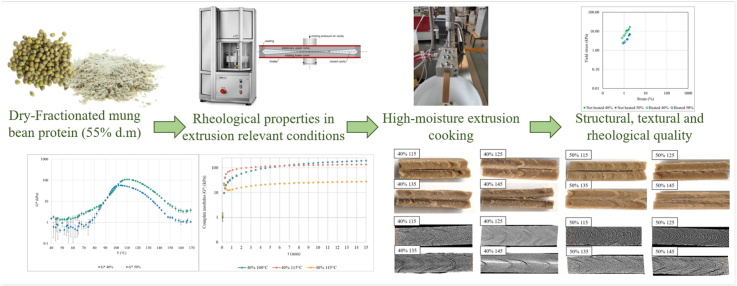

## 1 Introduction

Plant-based meat analogues are one of the hot topics in food technology research, as demonstrated by the increasing fundings gathered by both industry and academics (Good Food Institute, 2021). Most of the protein isolates or concentrates used in the preparation of meat analogues are derived from soy, pea, or wheat gluten (Bohrer, 2019), and they are commonly produced by a series of extractions with water and chemicals, which are generally grouped as ‘wet extraction’ technologies (Schutyser et al., 2015; Schlangen et al., 2022). For example, the alkaline extraction followed by the isoelectric precipitation is a complex process composed of several unit operations, which make it highly resource and energy demanding (Schutyser et al., 2015).

The environmental impact of the protein isolates/concentrates, indeed, is directly related to the extraction procedures (Lie-Piang et al., 2021). Therefore, from a long-term perspective, it is important to study how to introduce in the food system proteins obtained by sustainable processes, which are able to valorize the whole plant material. With this respect, the dry fractionation process is a sustainable alternative to the wet extraction, as it solely consists in a physical concentration of the protein by air classification, without using any water or chemicals. The operating principles of the dry fractionation are comprehensively described in previous works (De Angelis et al., 2021; Schutyser et al., 2015). In general, dry fractionation allows to produce a less refined protein concentrate, with a lower environmental footprint compared to the wet-extracted proteins (Vogelsang-O’Dwyer et al., 2020; Lie-Piang et al., 2021). Moreover dry-fractionated proteins are characterized by a complex chemical composition with starch, lipids, fibers and minerals alongside proteins, with consequent benefits in terms of some functional properties such as the solubility, the foaming activity (Vogelsang-O’Dwyer et al., 2020) and gelling behavior (Kornet et al., 2021). The latter is particularly relevant for the production of meat analogues (Schlangen et al., 2022). Despite this, there are few studies regarding the utilization of dry-fractionated proteins for meat analogues preparation. For example, in our previous research (De Angelis et al., 2020) the effectiveness of dry-fractionated pea and oat mixes has been evaluated in comparison with pea and soy isolates using the low-moisture extrusion cooking, whereas do Carmo et al. (2021) produced meat analogues by high-moisture extrusion using dry-fractionated faba bean protein with 63.5% protein content. Overall, these studies highlight the possibility to explore other protein sources for meat analogues applications, extending the range of ingredients available to produce meat alternatives.

For instance, mung bean (*Vigna radiata* (L.) R. Wilczek) is traditionally cultivated in Asia, Central Africa, America and Australia (Dahiya et al., 2015). Mung bean protein isolates are characterized by good functional properties, especially in terms of foaming and gelling behavior (Shrestha et al., 2022) and they are currently used in the food industry for the preparation of an egg substitute (Bansal-Mutalik et al., 2019 - Patent US10321705B2), whereas previous authors optimized the extrusion conditions for the development of texturized mung bean protein isolate (Brishti et al., 2021). Recently, the dry fractionation to obtain mung bean protein concentrate has been also proposed (Schlangen et al., 2022), highlighting the interest on this species.

Therefore, the aim of this paper is to evaluate the effectiveness of mung bean protein concentrate produced by dry fractionation for the preparation of meat analogues. To reach this goal, the rheological properties of the protein concentrate were evaluated by a closed cavity rheometer with the aim of studying the reaction behavior under elevated thermal and mechanical stresses, which imitate the extrusion relevant conditions (Emin et al., 2017; Pietsch et al., 2019; Schreuders et al., 2021b). Then, a high-moisture extrusion cooking process was carried out, setting four different barrel temperatures and two different moisture contents. The properties of the extrudates were then evaluated by observing their microstructure, and analyzing their texture, and their rheological properties.

## 2 Material and methods

### 2.1 Materials

Dry-fractionated mung bean protein concentrate –DFMB– was kindly supplied by Innovaprot srl (Gravina in Puglia, Italy). It was produced from whole mung bean seeds, that were finely milled with a pin mill (CW 250 II, Hosokawa Alpine AG, Augsburg, Germany) and fractionated using a turboplex ATP 315 air classifier (Hosokawa Alpine AG, Augsburg, Germany). DFMB had a protein content of 55 ± 1 g/100 g on dry matter basis, according to the manufacturer.

### 2.2 Rheological properties of the protein concentrate

Mung bean protein concentrate was vigorously mixed with water to reach two different moisture contents (40 and 50% v/w) and the mixes were then stored at 4 °C for at least 12 h to ensure a complete rehydration of the powders. For the rheological analysis, a Closed Cavity Rheometer (CCR from RPA Elite, TA Instruments, New Castle, Delaware, USA) was used. The structure and the working principle of the rheometer are carefully described in a previous work (Emin et al., 2017).

For the analysis, 5.5 g of the rehydrated protein concentrate were loaded between the two cones of the rheometer, and were submitted to defined thermal and mechanical stresses. The temperature sweep analysis was carried out by increasing the temperature from 40 to 170 °C at a rate of 5 K/min, angular frequency 1 Hz and strain amplitude 5%, kept constant during the analysis.

The time sweep analysis was carried out at a constant temperature of 100, 115 and 145 °C without applying any shear (angular frequency 1 Hz and strain amplitude 5%) and in high-shear conditions (angular frequency 10 Hz and strain amplitude 80%). The complex shear modulus G* was recorded as a function of the set conditions. All the determinations were repeated three times.

### 2.3 High-moisture extrusion cooking

The high-moisture extrusion cooking was carried out with a co-rotating twin-screw extruder (Process 11, ThermoFisher Scientific Inc., Waltham, MA, USA) with a length to diameter ratio (L/D) of 40 and a screw diameter of 11 mm. Cooling die (125 × 19 × 4 mm L × W × H) was cooled at a T of 10 °C.

The extruder barrel consisted of seven heating elements. A gravimetrically controlled feeder (Brabender Technology GmbH, Duisburg, Germany) was used to dose the protein powder, whereas the water content was dosed using a peristaltic pump (Masterflex L/S, Cole Parmer, Vernon Hills, IL, USA). The total mass flow rate (solids + water) was kept constant at 0.7 kg/h. This flow rate was determined in preliminary tests, and it was able to guarantee stable conditions during the whole extrusion process.

The moisture content of the melt was set to 40% and 50% (w/w) in order to work under high-moisture extrusion conditions (Palanisamy et al., 2019; Wittek et al., 2021b) and having a correspondence between the moisture content used in the rheological analysis of the protein and the moisture of the melt during the extrusion trials. The water pump and the gravimetric feeder were calibrated prior the extrusion experiment to ensure the correct moisture content of the melt. For each moisture content, four temperatures were used, i.e., 115, 125, 135 and 145 °C, controlling the last three zones of the extruder barrel. The screw speed was kept constant at 800 rpm for all processing conditions, and it was chosen after preliminary trials, also considering that dry-fractionated proteins are usually processed with high screw speed (De Angelis et al., 2020; do Carmo et al., 2021). The extrusion conditions used in this study are reported in Table 1. The pressure was monitored with a pressure sensor.

**Table 1:** Process conditions used for the extrusion experiments. Temperatures are reported for the different heating zones of the extruder.

Trials	Moisture content (%)	Screw speed (rpm)	Feed rate (kg/h)	T1 (°C)	T2 (°C)	T3 (°C)	T4 (°C)	T5-7 (°C)
1	40	800	0.7	20	40	60	80	115
2	40	800	0.7	20	40	60	80	125
3	40	800	0.7	20	40	60	80	135
4	40	800	0.7	20	40	60	80	145
5	50	800	0.7	20	40	60	80	115
6	50	800	0.7	20	40	60	80	125
7	50	800	0.7	20	40	60	80	135
8	50	800	0.7	20	40	60	80	145

### 2.4 Visualization of the anisotropic structure of the extrudates

The structure of the extrudates was visually observed immediately after extrusion processing by cutting and opening the samples along the flow direction. Moreover, the anisotropic structure was visualized by the X-ray micro computed tomography (micro-CT), with the procedure described in Kendler et al. (2021). In particular, the samples were cut in 8 cm length pieces and freeze-dried in an Alpha 1–4 LDplus laboratory freeze dryer (Martin Christ Gefriertrocknungsanlagen GmbH, Osterode, Germany). The freeze-dried samples were analyzed in the micro-CT Xradia 520 (Carl Zeiss Microscopy GmbH, Oberkochen, Germany). Digital image processing software (ImageJ software version 1.50 d, National Institutes of Health, Bethesda, MD, USA) was used for noise filtering and segmentation (Kendler et al., 2021).

### 2.5 Textural and rheological properties of the extrudates

The textural properties were evaluated by a ZI.0 TN texture analyzer (ZwickRoell GmbH & Co. KG, Ulm, Germany), equipped with 1 kN load-cell and a compression probe of 36 mm diameter, using the conditions described in De Angelis et al. (2020) with few modifications. The extrudates were cut in pieces of 19 × 19 × 4 mm (L × w × h) and compressed twice until 75% of deformation, at speed of 1 mm/s with 5 s of break within the two compressions. The analysis was repeated five times.

The extrudates were subjected to an amplitude sweep analysis, carried out with the closed cavity rheometer according to the procedures described in Schreuders et al. (2022). The analysis was performed at two different conditions: i) extrudates heated at 30 °C for 2 min without any shear and then analyzed with a constant frequency of 1 Hz and a strain γ = 0.1–1000%; ii) extrudates heated at 65 °C for 2 min and cooled to 30 °C at a rate of 5 °C/min without applying any shear. Once cooled down at 30 °C, they were analyzed with a constant frequency of 1 Hz and a strain γ = 0.1–1000%. The rheological analyses were repeated three times.

### 2.6 Statistical analysis

Data were subjected to one-way analysis of variance (ANOVA) followed by Tukey's honestly significant difference test for multiple comparisons at a significance level α = 0.05 using Minitab 19 (Minitab Inc., State College, PA, USA, USA).

## 3 Results and discussion

### 3.1 Rheological properties of the protein

**Fig. 1:**
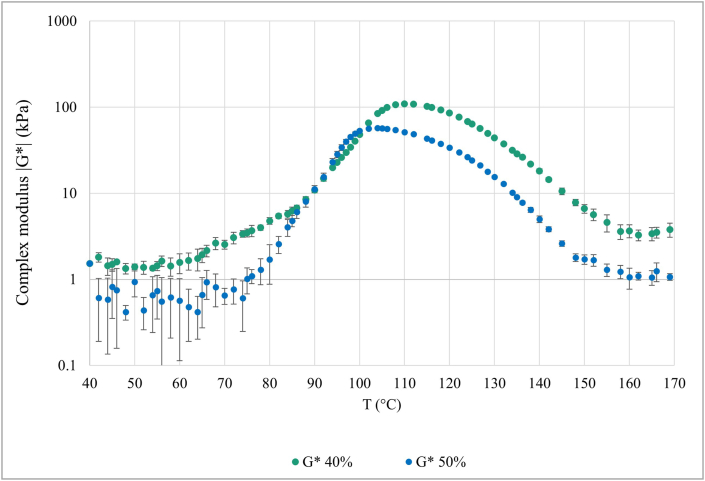
Change in the complex modulus (G*) as a function of increasing temperature of the dry-fractionated mung bean protein prepared at water content of 40% and 50%.

**Fig. 2:**
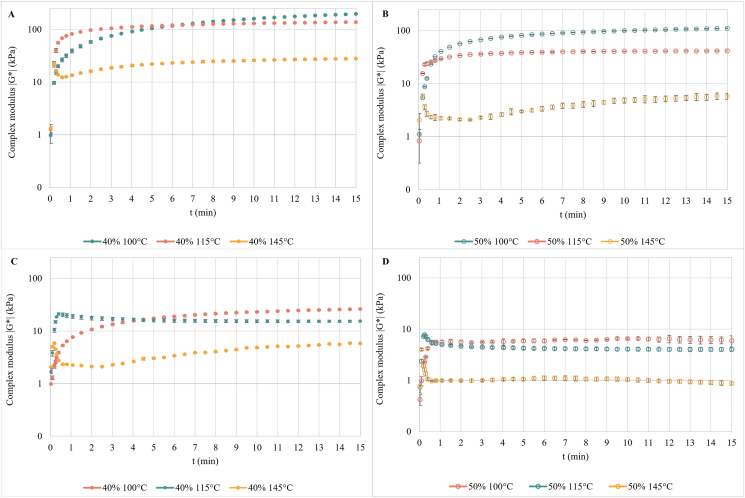
Change in the complex modulus (G*) of the dry-fractionated mung bean protein prepared at water content of 40% and 50% as a function of time in isothermal conditions: without shear at angular frequency 1 Hz and strain amplitude 5% (A, B), with shear at angular frequency 10 Hz and strain amplitude 80% (C, D).

The rheological properties of the DFMB protein prepared at 40% and 50% of moisture content are shown in Fig. 1. Both the protein mixes were characterized by a low complex modulus (G*) at the beginning of the analysis, at a temperature range between 30 and about 70 °C. The G* was lower compared to wet extracted protein such as wheat gluten (Emin et al., 2017) and soy protein (Jia et al., 2021; Schreuders et al., 2021a) prepared with the same or even higher moisture content. This behavior can be related to the water absorption capacity of the dry-fractionated proteins, which is generally lower compared to the proteins produced by wet extraction technologies (De Angelis et al., 2020; Vogelsang-O’Dwyer et al., 2020). This occurs because dry-fractionated proteins are in their native state and tend to remain soluble in water rather than binding it (De Angelis et al., 2020; Vogelsang-O’Dwyer et al., 2020). An increase of the G* was observed starting from about 70 °C. It is reasonable to assume that the increase in the complex modulus is related to both the starch gelatinization and the protein gelation. In particular, mung bean starch gelatinization starts at temperatures above 60 °C, and it is concluded at temperatures of 75–82 °C (Hoover et al., 2010). This hypothesis was corroborated by the findings previously reported in Schlangen et al. (2022) and Kornet et al. (2021). At temperatures higher than 75 °C the protein starts to denature, and to interact leading to the gelation process (Schlangen et al., 2022), causing a more pronounced increase of the G*, due to the formation of links between the protein molecules. The increase of the complex modulus continues with the increase of the temperature, reaching a peak at about 105 °C and 110 °C for the protein at 50% and 40% moisture content, respectively, which can indicate the end of the aggregation reactions. This trend was previously observed in wheat gluten (Emin et al., 2017), who reported a faster reaction rate given by a higher molecular mobility. Then, the G* started to decrease and this may indicate the onset of the degradation reactions which cause the breakdown of the formed structure. However, further insights on this speculation are given with the comment of Fig. 2.

The G* decreasing rate appears to be similar between the protein prepared at 50% and 40% moisture content. Overall, the DFMB protein showed a reaction behavior very similar to the wheat gluten, which is known to be native (Emin et al., 2017). By contrast, the rapeseed and the soy protein isolates produced by wet extraction showed a decreasing G* when subjected to a temperature sweep (Jia et al., 2021). In fact, in the dry-fractionated proteins the structure of the protein is native, whereas in the wet-extracted ones it tends to be denatured (Schutyser et al., 2015; Vogelsang-O’Dwyer et al., 2020), leading to a different behavior during the heating process.

To better understand the rheological behavior of the DFMB protein, the evolutions of G* as a function of time at constant temperatures of 100, 115 and 145 °C, and at 40% and 50% water contents, are shown in Fig. 2. The time-dependent reaction behavior was determined at two different shear rates. In no-shear conditions (shear rate 0.3 s^−1^), the protein mix is analyzed in the linear viscoelastic region and the mechanical stress on the sample is not relevant. In such conditions, the behavior of the DFMB protein seems mainly affected by the temperature and not by the moisture content, which only causes a change in the absolute values of the complex modulus G* (Fig. 2a and b). G* slowly increases at 100 °C without reaching a peak over the time studied, indicating that structure-forming reactions are still taking place at this temperature for the time considered. The same happened at 115 °C, but at this temperature the rate of increase was faster, especially in the first minute of analysis, than what was observed at 100 °C. According to the temperature sweep analysis (Fig. 1) at such temperatures the structure formation and the polymerization reactions were still ongoing, since this is the region near the peak of the curve. At 145 °C the complex modulus showed the lowest values, corroborating what was observed in the temperature sweep (Fig. 1). At this temperature, a small peak in the first seconds of analysis was observed, and it can be related to the fast heating of the sample from room temperature to the operating temperature (Schreuders et al., 2020). However, it is interesting to highlight that, in contrast with what was expected by observing Fig. 1, G* did not decrease over time at 145 °C, showing instead a slight increase. This trend was not previously found when other protein sources, such as pea isolate (Schreuders et al., 2020), rapeseed and soy (Jia et al., 2021), and wheat gluten (Emin et al., 2017) were studied at high temperatures. The cause could be the composition of the protein used. Indeed, the mentioned studies are referred to highly pure proteins. Then, it might be speculated that other components of the DFMB protein, such as carbohydrates (Pietsch et al., 2019) and fibers, may interact at this temperature, leading to the slight increase in G*. Moreover, it could be reasonable to assume that the major changes occurring at the G* with increasing temperatures might be principally related to a higher molecular mobility of the protein, and not to the depolymerization reactions (Wittek et al., 2021a, 2021b). Such aspects could be more deeply addressed in future research on the reaction behavior of dry-fractionated proteins.

In high-shear conditions, i.e. shear rate of 50 s^−1^ (Fig. 2 c and d), it is possible to highlight the decrease of the G* absolute values, indicating the shear thinning behavior of the DFMB protein. The trend observed for the three temperatures and the two moisture contents was very similar to what was noticed in no-shear conditions. Overall, this may indicate that dry-fractionated protein could be used for the texturization during the high-moisture extrusion cooking in a wide range of temperatures with different moisture contents.

### 3.2 High-moisture extrusion cooking and structure of the extrudates

**Fig. 3:**
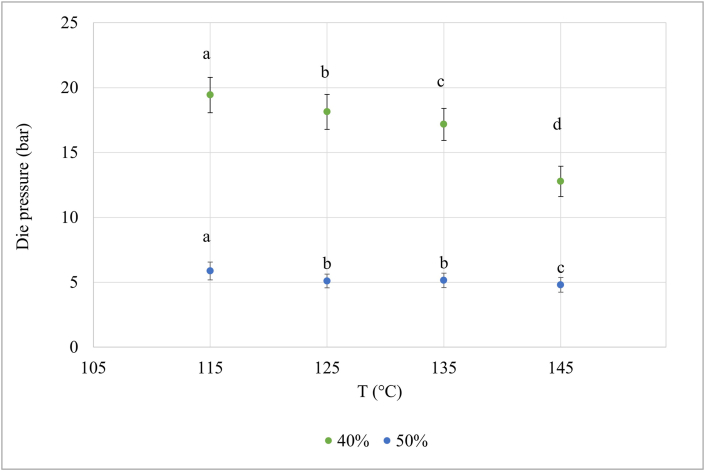
Change in the pressure (bar) as a function of increasing temperature and water content of 40% and 50%. Different letters mean significant differences according to the one-way ANOVA at α = 0.05 followed by the Tukey's test.

Fig. 3 depicts the mean die pressure recorded during the extrusion process as a function of the barrel temperature at a constant mass flow rate. The extruder pressure indicates major changes in the process conditions, and it is representative of variations in the melt viscosity (Pietsch et al., 2019). The effect of the temperature on the die pressure was significant, and an increase in temperature led to a decrease of the die pressure in both 40% and 50% moisture content processes. These findings indicate a reduction of the melt viscosity that can be attributed to an increment of the molecular mobility given by the high temperature (Pietsch et al., 2019; Wittek et al., 2021b). Specifically, the protein dough develops into a liquid-like matter under high temperatures and shear conditions, which causes a weakening of the intermolecular bonds (van der Sman and van der Goot, 2023), and consequently a temperature-dependent drop in the viscosity. Moreover, the effect of the polysaccharides still contained in the dry-fractionated proteins (De Angelis et al., 2021) cannot be considered as negligible. In fact, the degradation of starch and other polysaccharides components under thermomechanical treatments may contribute to the decrease in the melt viscosity (Pietsch et al., 2019; van der Sman and van der Goot, 2023).

Moreover, these changes are consistent with the results of the rheological analysis of the protein. For instance, the complex modulus decreased with increasing temperatures, as observed in the temperature sweep at temperatures above 110 °C (Fig. 1) and in the time sweep (Fig. 2).

The effect of the temperature on the die pressure is dependent on the protein type. In fact, a reduction of the pressure at increasing temperatures was previously reported for soy (Pietsch et al., 2019) and lupin protein (Palanisamy et al., 2019). By contrast, an opposite behavior was found during the extrusion of the wheat gluten apparently due to the polymerization reactions, which is assumed to occur in this protein at high temperatures (Pietsch et al., 2017).

**Fig. 4:**
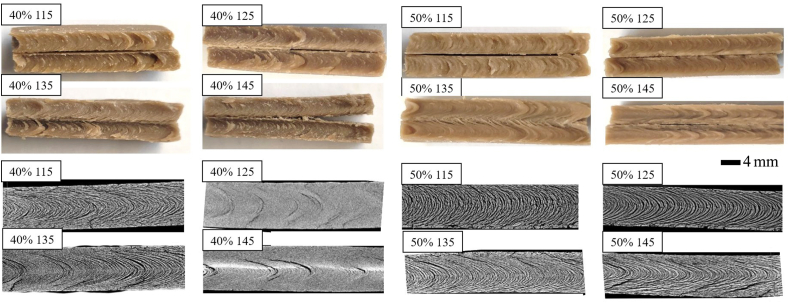
Visual appearance of the fracture profile (top) and micro-computed tomography images (bottom) of dry-fractionated mung bean protein concentrate extruded at different temperatures and moisture contents. The flow direction is from the left to the right.

The fracture profile of the extrudates together with the micro-CT images are shown in Fig. 4. They are useful to visualize and compare the anisotropic structure of the product. All samples showed a distinct V-shaped fracture profile, which originates from the laminar flow in the cooling die. The fracture profile appears to be influenced by the extrusion temperature, and this effect was especially visible at 50% moisture content, whereas it was less evident in the extrudates produced at 40% moisture content. In particular, it can be noted that higher temperature led to a more pointed V-profile and more pronounced alignment of the protein matrix into the flow direction, making the formation of the anisotropic structure more evident in the meat analogues produced at 145 °C than at 115 °C. The micro-CT better highlighted the changes induced by the different temperatures, pointing out that this technique can provide useful information for the analysis of the anisotropic structure of the meat analogues. It was reported that higher extruder temperatures promote the formation of molecular cross-links between proteins (Chen et al., 2010; Palanisamy et al., 2019). More in depth, according to recent hypothesis on the formation of the anisotropic structure, all the intermolecular bonds between proteins and polysaccharides, e.g., hydrogen bonds, hydrophobic interactions, salt bridges, and disulfide bonds, can be subjected to continuous breaking and forming reactions under high temperature and high shear conditions (van der Sman and van der Goot, 2023). Then, this transient network is stabilized in the cooling die and the bonds become more durable, affecting the rheological properties of the material (van der Sman and van der Goot, 2023). Therefore, the different formation of the anisotropic structure can be related to the lower viscosity of the melt at higher temperature (Fig. 3), also confirming what was previously found by Pietsch et al. (2019) and Wittek et al. (2021a). We can corroborate that the elongation of the material is favored at high temperatures, due to reduced shear stresses in the cooling die. Consequently, an important contribution to the formation of the anisotropic structure is also given by the moisture content as the viscosity decreases with increasing water content. In particular, as water has a plasticizer effect, the water content can modulate the formation of intermolecular bonds. It is known that higher moisture content can promote more disulfide and hydrogen bonds (van der Sman and van der Goot, 2023), supporting the fibers alignment (Chen et al., 2010).

Overall, it is interesting to highlight that it would be possible to produce meat analogues with very different fibrous structure by simply changing the moisture content and the temperature during the process. Consequently, the textural and the rheological properties of the extrudates may also be affected, as discussed in section 3.3.

### 3.3 Rheological and textural properties of the extrudates

**Fig. 5:**
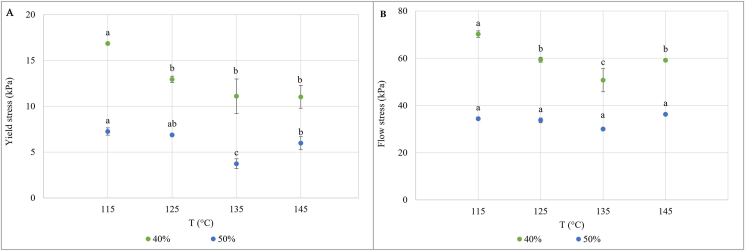
Change in the yield stress and flow stress of the extrudates of dry-fractionated mung bean protein concentrate as a function of increasing temperature and at water content of 40% and 50%. Different letters for the same moisture content mean significant differences according to the one-way ANOVA at α = 0.05 followed by the Tukey's test.

The yield stress of the meat analogues produced at different temperatures and moisture content is shown in Fig. 5. The yield stress is defined as the value of the shear stress (kPa) at the end of the linear viscoelastic region (Schreuders et al., 2022), beyond which the material starts to breakdown (McClements et al., 2021). This stress is calculated as the point where the G′ differs more than 5% from its initial value (Schreuders et al., 2022). The yield stress was the highest in the meat analogues produced at 115 °C, and then, it tended to decrease at increasing temperatures. The moisture content only led to a reduction of the yield stress absolute values, while the observed trend was similar between the meat analogues obtained at 40% and 50% moisture contents. The overall reduction of the yield stress at higher temperatures may be correlated with what was observed in the temperature sweep analysis (Fig. 2), in which a progressive reduction of the complex modulus at increased temperature was detected. A lower yield stress indicates that the product is less able to withstand deformations and that a lower stress is required to disrupt the structure of the sample. Indeed, the amplitude sweep analysis was carried out directly on the extrudates to understand their behavior and their resistance under defined stress and to quantify the structural strength of the product (Pietsch et al., 2021). A very similar behavior was also found for the flow stress (i.e., the shear stress calculated at the crossover point, when G′ is equal to G″) (Schreuders et al., 2022), which showed a positive and significant correlation with the yield stress (R = 0.966, *p* < 0.001).

The rheological evaluation of the structural properties of meat analogues was previously proposed by few authors, such as Mattice and Marangoni (2020), Pietsch et al. (2021), and Schreuders et al. (2022), on zein-structured products, soy and gluten extrudates, and commercial meat analogues, respectively. Therefore, the comparison of the results is quite challenging since the authors did not evaluate the effect of different extrusion conditions on the yield stress and/or the flow stress.

The results of the texture profile analysis of the meat analogues are reported in Table 2, with the significant differences calculated within the same moisture content. The textural properties of the meat analogues significantly differed, especially for the hardness, the chewiness, and the cohesion. Interestingly, increasing the temperature during extrusion led to the production of meat analogues characterized by a higher hardness, chewiness and cohesion, regardless the moisture content. Such behavior was unexpected considering the results of the rheological evaluation (Fig. 5), which overall described the product obtained at higher temperature as less able to withstand deformations. However, the positive influence of the temperature on the firmness and tensile strength of meat analogues was previously reported (Chen et al., 2010; Osen et al., 2014). In particular, the observed behavior can be related to the more pronounced anisotropic structure seen in the product obtained at higher temperatures (Fig. 4), corroborating previous findings reporting that a higher degree of texturization was responsible of more consistent products (Osen et al., 2014; Palanisamy et al., 2019; Pietsch et al., 2019). Moreover, it should be reminded that the texture evaluation only causes a longitudinal deformation of the sample, whereas the rheological evaluation was carried out in a continuous oscillation at increasing amplitude. Therefore, a combination of both the analytical determinations can offer an overall overview of the structural characteristics of the meat analogues.

**Table 2:** Results of the texture profile analysis of the meat analogues produced at different temperature and moisture contents.

Sample	Hardness (N)	Springiness	Chewiness (N)	Cohesion
40% 115	369 ± 14^c^	0.70 ± 0.02^a^	133±8^c^	93±7^b^
40% 125	402±9^b^	0.52 ± 0.08^a^	171±1^b^	88 ± 14^b^
40% 135	434±6^a^	0.68 ± 0.01^a^	188±2^a^	128±0^a^
40% 145	446 ± 12^a^	0.59 ± 0.25^a^	195±5^a^	140 ± 13^a^
50% 115	151 ± 10^c^	0.80 ± 0.08^a^	46±6^d^	36±4^c^
50% 125	159±5^c^	0.75 ± 0.04^a^	57±2^c^	43±2^c^
50% 135	260 ± 10^b^	0.72 ± 0.07^a^	129±7^b^	94 ± 12^b^
50% 145	286±3^a^	0.75 ± 0.01^a^	145±1^a^	109±2^a^

Different letters for the same parameter mean significant differences within the same moisture content according to the one-way ANOVA at α = 0.05 followed by the Tukey's test.

**Fig. 6:**
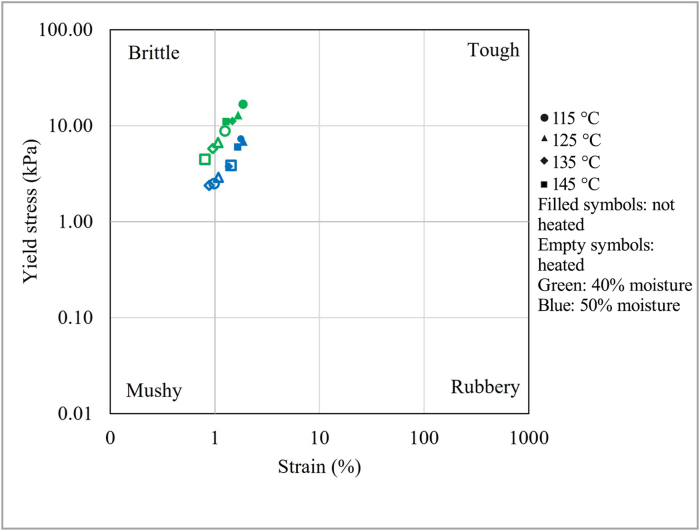
Texture map at the end of the linear viscoelastic region of the products analyzed at 30 °C, and heated at 65 °C and then analyzed at 30 °C.

To understand if the product structure was affected by further processing, i.e., heating, the samples were heated up to 65 °C, cooled down to 30 °C and then analyzed (Schreuders et al., 2022). Such procedure would simulate the service conditions of the meat analogues, and it can be compared to the last preparation step before food consumption. The yield stress and the critical strain are plotted in Fig. 6 to create a texture map (Schreuders et al., 2021b). In particular, the figure can be divided in four quadrants, carefully explained by Schlangen et al., 2022, Schreuders et al., 2021b, Schreuders et al., 2022. In the bottom-left, at low yield stress and low shear strain, the samples can be classified as a soft, non-shaped texture (similar to porridge or similar products). In the bottom-right, the samples have low yield stress and high shear strain, and they are often described as “rubbery” (e.g., gelatin). In the top-right of Fig. 6 the samples have a “tough” texture indicating a hard-to-break material with high yield stress and shear strain. Finally, in the top-left, the samples are characterized by a “brittle” texture (e.g., bakery products), having high yield stress but low shear strain.

According to their yield stress and critical strain, the unheated meat analogues are located near the top-right quadrant, indicating their tendency to be brittle rather than tough. To have a better idea of the product characteristics, Schreuders et al. (2022) reported that different unheated commercial plant-based meat analogues were also located near the top-left and top-middle quadrants, indicating that the meat analogues obtained by the dry-fractionated protein showed rheological properties similar to that of the commercial ones produced with soy protein and/or wheat gluten. Unheated pork and beef meet, analyzed with the same protocol, were reported to be in the middle of the graph (Schreuders et al., 2022).

The heating process led to a slight shifting of the samples toward the bottom-left quadrant, which means that the products become softer. The same behavior was reported in Schreuders et al. (2022) and it can be related to the effect of weakening of the physical interactions that stabilize the protein material (Schreuders et al., 2021b). Interestingly, in the study of Schreuders et al. (2022), meat products showed an opposite behavior, shifting to the right, corresponding to them becoming tougher. This trend was explained by the heat-denaturation of myofibrillar proteins (Schreuders et al., 2022).

## 4 Conclusions

The research on plant-based meat analogues is focused on the utilization of protein isolates with a high-protein content, whereas the dry-fractionated proteins are still poorly employed in this field. In this research, the potential applicability of the dry-fractionated mung bean protein for the texturization process was investigated using a rheological approach aimed at the evaluation of the protein reaction behavior under extrusion relevant conditions.

In particular, the DFMB protein showed a reaction behavior linked to the native state of the protein and characterized by the predominance of the aggregation reactions until 105 °C and 110 °C for the protein at 50% and 40% moisture content, respectively. The time sweep analysis highlighted that the effect of the temperatures on the behavior of the protein was predominant, whereas the moisture content led to decreases only in the G*absolute values. However, future works may better investigate the structure formation of dry-fractionated proteins in terms of type of bonds formed during the thermal treatments.

The high-moisture extrusion was carried out within the temperatures and moisture content studied during the rheological analysis of the protein, in order to explain some of the phenomena observed in the extrudates, by the rheological data. For instance, increasing the extruder temperature led to a reduction of the die pressure, and consequently of the melt viscosity. This effect was also visible in the images of the microstructure of the extrudates, which showed a more pointed V-shaped flow profile and a more pronounced anisotropic structure when higher temperatures were applied. The textural indices of the extrudates i.e., hardness, chewiness and cohesion were directly correlated with the temperature. However, this was contrasting with the results of the rheological evaluation which described the product obtained at higher temperature as less able to withstand deformations. Therefore, integrated approaches are needed for a comprehensive evaluation of the structural properties of the meat analogues. The overall evaluation of the meat analogues through the texture map, highlighted similarities with some commercial meat analogues, and indicated that working on the process conditions and on the ingredients, it would be possible to produce meat analogues with different structural properties from dry fractionated mung bean.

## Funding

This research did not receive any specific grant from funding agencies in the public, commercial, or not-for-profit sectors.

## CRediT authorship contribution statement

**Davide De Angelis:** Conceptualization, Investigation, Formal analysis, Writing – original draft, preparation, Writing – review & editing. **Christina Opaluwa:** Conceptualization, Investigation, Writing – original draft, preparation. **Antonella Pasqualone:** Project administration, Resources, Writing – review & editing. **Heike P. Karbstein:** Conceptualization, Resources, Project administration, Writing – review & editing. **Carmine Summo:** Conceptualization, Resources, Project administration, Writing – review & editing.

## Declaration of competing interest

The authors declare that they have no known competing financial interests or personal relationships that could have appeared to influence the work reported in this paper.

## Data Availability

Data will be made available on request.
